# The Role of Dietary Polyphenols in Pregnancy and Pregnancy-Related Disorders

**DOI:** 10.3390/nu14245246

**Published:** 2022-12-09

**Authors:** Mirjana Nacka-Aleksić, Andrea Pirković, Aleksandra Vilotić, Žanka Bojić-Trbojević, Milica Jovanović Krivokuća, Francesca Giampieri, Maurizio Battino, Dragana Dekanski

**Affiliations:** 1 Institute for the Application of Nuclear Energy, Department for Biology of Reproduction, University of Belgrade, Banatska 31b, 11080 Belgrade, Serbia; 2 Research Group on Food, Nutritional Biochemistry and Health, Universidad Europea del Atlántico, 39011 Santander, Spain; 3 Department of Biochemistry, Faculty of Sciences, King Abdulaziz University, Jeddah 21589, Saudi Arabia; 4 International Joint Research Laboratory of Intelligent Agriculture and Agri-Products Processing, Jiangsu University, Zhenjiang 212013, China; 5 Dipartimento di Scienze Cliniche Specialistiche, Facoltà di Medicina, Università Politecnica delle Marche, 60131 Ancona, Italy

**Keywords:** polyphenols, early pregnancy, trophoblast, gestational diabetes mellitus, preeclampsia

## Abstract

Polyphenols are a group of phytochemicals with extensive biological functions and health-promoting potential. These compounds are present in most foods of plant origin and their increased widespread availability through the intake of nutritional supplements, fortified foods, and beverages, has also led to increased exposure throughout gestation. In this narrative review, we focus on the role of polyphenols in both healthy and pathological pregnancy. General information related to their classification and function is followed by an overview of their known effects in early-pregnancy events, including the current insights into molecular mechanisms involved. Further, we provide an overview of their involvement in some of the most common pregnancy-associated pathological conditions, such as preeclampsia and gestational diabetes mellitus. Additionally, we also discuss the estimated possible risk of polyphenol consumption on pregnancy outcomes. The consumption of dietary polyphenols during pregnancy needs particular attention considering the possible effects of polyphenols on the mechanisms involved in maternal adaptation and fetal development. Further studies are strongly needed to unravel the in vivo effects of polyphenol metabolites during pregnancy, as well as their role on advanced maternal age, prenatal nutrition, and metabolic risk of the offspring.

## 1. Introduction

**Figure 1:**
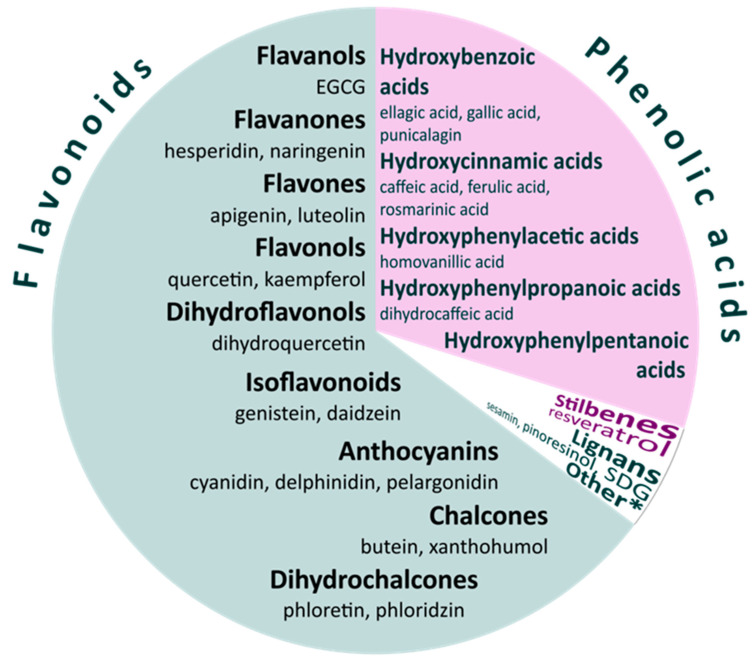
Main classes and subclasses of dietary polyphenols. Flavonoids make approximately two-thirds of total polyphenol intake; phenolic acids account for the remaining one-third. * Other polyphenols are alkylmethoxyphenols, alkylphenols, curcuminoids (curcumin), furanocoumarins (psoralen), hydroxybenzaldehydes (vanillin), hydroxybenzoketones, hydroxycinnamaldehydes, hydroxycoumarins (coumarin, esculetin), hydroxyphenylpropenes (eugenol), methoxyphenols, naphtoquinones, phenolic terpenes (carvacrol, thymol), tyrosols (hydroxytyrosol, tyrosol, oleuropein). EGCG—epigallocatechin-gallate; SDG—secoisolariciresinol diglucoside.

Dietary polyphenols (also known as phenolics) are a heterogeneous group of more than 8000 different biologically active compounds in plant-based foods. They are the major group of non-nutrients and the most abundant dietary antioxidants [1]. The main dietary sources of polyphenols are fruit and beverages (fruit juice, wine, tea, coffee, chocolate, and beer) and, to a lesser extent vegetables, dry legumes, and cereals [2]. They are classified on the basis of the number of phenol rings and of the structural elements that bind these rings to one another. According to Phenol-Explorer, the most complete and most widely used database in European countries [3,4], the major groups are flavonoids, phenolic acids, stilbenes, and lignans (Figure 1). Among these, flavonoids are the most abundant polyphenols in the diet and can be subcategorized as anthocyanins, chalcones, dihydrochalcones, dihydroflavonols, flavanols, flavanones, flavones, flavonols, and isoflavonoids [5]. In most cases, foods contain complex mixtures of polyphenols. Certain polyphenols such as the flavonol quercetin are found in almost all plant products, whereas flavanones (naringenin) and isoflavonoids (genistein) are specific to particular foods [6]. Flavonoids account for about two-thirds of the total intake, while phenolic acids (hydroxycinnamic acids and hydroxybenzoic acids) account for the remaining one-third. Lignans (sesamin) and stilbenes (resveratrol) are far less common in the human diet [2].

However, the polyphenols that are most commonly consumed in our diets are not necessarily the most bioavailable due to inefficient absorption or rapid excretion [7]. It is important to stress that the chemical structure of polyphenol (not its concentration) determines the rate and extent of absorption and the nature of the metabolites circulating in the plasma. After ingestion, glycosylated polyphenols are hydrolyzed by intestinal enzymes prior to being absorbed through the intestinal wall. Nonglycosylated polyphenols are absorbed in the small intestine by passive diffusion. Circulating polyphenols reach the liver and undergo extensive modification (methylation, sulfation, and/or glucuronidation). Following biotransformation, weakly conjugated polyphenols re-enter circulation and are excreted in the urine, while highly conjugated polyphenols are excreted in the bile and enter the large intestine, where they can be processed by the colonic microflora and then reabsorbed into circulation or excreted in the feces. Consequently, the forms of polyphenols reaching the blood and tissues are different from those present in food [6]. The maximum concentration in plasma rarely exceeds 1 µM after the consumption of 10–100 mg of a single phenolic compound. However, the total plasma phenol concentration is probably higher due to the presence of metabolites formed in the body’s tissues or by the colonic microflora [2]. In recent years, polyphenol metabolites have attracted great interest as many of them showed similar or higher intrinsic biological activities in comparison to the parent compounds [8,9]. Further, the effect of polyphenolic compounds in combination may be very different from those expected by each of these compounds alone [10]. The multitargeting and pleiotropic effects of polyphenols and their metabolites makes them considered as health-promoting compounds. In the scientific community, these micronutrients have attracted attention given the recent evidence of their role in the prevention of metabolic, cardiovascular, and neurodegenerative diseases, as well as in some types of cancer [11]. An inverse association has been determined between the consumption of foods rich in polyphenols and the risk of chronic noncommunicable diseases [12]. Namely, epidemiological studies have revealed that polyphenol consumption provides significant protection against the development of cardiovascular diseases, asthma, diabetes, cancer, tissue inflammations, aging, etc., thus indicating that the role of polyphenols in human health is still a fertile area of research [13,14,15,16,17,18,19,20]. However, in spite of the beneficial effects observed in various chronic diseases in humans, limited and inconclusive information is currently available about their effects in pregnant women. Maternal nutrition plays an important role in providing the necessary energy and nutrients for fetal growth and development. The growing interest in plant-derived substances has led to increased consumption of dietary polyphenols throughout pregnancy. The total average polyphenol daily intake is approximately 1 g [2]; however, the recent study in which dietary intake was measured in different populations was shown that the highest consumption of polyphenols (approx. 2 g daily) was observed in pregnant women [21]. Polyphenolic compounds are, to a varying extent, absorbed from the gut lumen into the blood circulation [6,7], and so the placenta will be exposed to these compounds. Low levels of polyphenols have been detected in the placenta [22], but it is important to highlight that bioavailability appears to differ greatly between the various polyphenols and that despite low oral bioavailability, most polyphenols showed significant biological effects [23]. Research data indicates that transport across the placenta is not efficient and that the placenta seems to act as a barrier for flavanols and their metabolites. However, these compounds target the fetus and are excreted in the amniotic fluid [24]. It is worth noting that polyphenol transportation through the placenta involves selective transport mechanisms, suggesting that these compounds are able to cross the placental barrier, and therefore may have biological effects on the offspring [22]. It is well established that polyphenols act as conventional hydrogen- or electron-donating antioxidants and consequently increase plasma antioxidant capacity and improve oxidative stress parameters at the fetoplacental unit, which are recognized as main issues in different pregnancy pathologies. In addition to their antioxidant properties, dietary polyphenols and their in vivo metabolites may exert modulatory actions in cells through actions at protein kinase and lipid kinase signaling pathways [25]. These compounds modulate the activity of a wide range of enzymes and cell receptors [26], and they also interfere with the activity and expression of several cell membrane transporters. Thus, the placenta is also a target for the action of polyphenols, which could interfere with the placental uptake of nutrients or other bioactive substances from the maternal circulation [10].

This review aims to present a wide spectrum of literature data and to summarize the recent findings on dietary polyphenol activity in pregnancy, obtained from in vitro studies, animal models, and clinical trials, including an insight into the molecular mechanisms involved. Bearing in mind their abundance in food and the wide repertoire of biological properties, the use of polyphenols could be a promising strategy for preventing or alleviating some of the complications in pregnancy. However, it should be noted that the diverging effects of polyphenols on health can lead to both beneficial and risk-inducing consequences for fetal health. Therefore, the inconclusive estimation of their benefits and safety will also be discussed.

## 2. The Role of Dietary Polyphenols in Early-Pregnancy Events

The formation of functional placenta is fundamental for pregnancy success, and depends on well-coordinated proliferation and differentiation of specialized trophoblast cells. The human placenta is composed of different trophoblast cell subpopulations that possess diverse functional characteristics [27]. Three major trophoblast subtypes are recognized in human placenta: syncytiotrophoblast (STB), cytotrophoblast (CTB), and extravillous trophoblast (EVT) cells [28]. During differentiation, CTB cells can function as stem cell–progenitor-like cells that can undergo either cell fusion, forming a layer of multinucleated STB cells, or epithelial mesenchymal transition, creating EVT lineage. Syncytiotrophoblasts are a main site of nutrient and gas exchange, as well as hormone production. On the other hand, EVTs are capable of invading maternal decidua and transforming spiral arteries, which is essential for placental and fetal development [28]. In early-pregnancy events, low oxygen environment has been recognized as one of the crucial regulatory factors [29,30]. Thus far, studies in vivo have provided evidence that the fetus and first-trimester placenta develop in relative hypoxia [30]. Furthermore, low oxygen level is also detected in intervillous space. Despite that, during this period, the placenta grows significantly. The progression of pregnancy oxygen level, from 2–4% O_2_ measured at 8 weeks of pregnancy to 10% at 12 weeks, triggers oxidative stress (OS) in trophoblasts [29]. This rise is associated with the increases in mRNAs and activities of the antioxidant enzymes catalase, copper/zinc superoxide dismutase, and glutathione peroxidase in placenta tissue [29]. Together with this, maternal blood enters into the intervillous space. These highly controlled events appear at the placental periphery and spread to the center, thus protecting the fetus from sudden OS. The overwhelming evidence has indicated that OS is implicated in angiogenesis, proliferation, differentiation, and invasion of the trophoblast during placentation [31]. As shown by a study in vitro, hypoxia affects CTBs’ cell fate by changing the balance between proliferation and differentiation of CTBs [32]. Hypoxia, which mimics the situation in vivo, induces CTBs to enter mitosis, reducing their invasiveness, while higher oxygen tension triggers CTBs’ differentiation. It has been observed that hypoxic conditions selectively affect the expression of molecules required for trophoblast invasion. For example, when culturing in low oxygen condition, CTBs express integrins and molecules of the extracellular matrix (α5 and β1 integrins and fibronectin) responsible for early stages of differentiation (before the 7th gestation week), but fail to express α1 integrin, which appears during the later invasion phase [33]. This is in line with the results, which show that hypoxic conditions inhibit trophoblast invasion at ~10 weeks of gestation [33]. Clearly, oxygen influences gene regulation and downstream events in the placenta, but excessive OS could harm placental homeostasis and seems to be associated with pregnancy-related disorders such as early pregnancy loss, gestational diabetes mellitus (GDM), preeclampsia (PE), and intrauterine growth restriction (IUGR). Thus, maintaining the balance between a proper placentation and redox system is one of the key points during gestation. In that context, understanding the influence of dietary polyphenols as strong antioxidants, which are consumed during pregnancy, is of great importance. Although their effect on pregnancy is mainly considered as beneficial and is attributed to their antioxidant properties, increasing data also support their anti-inflammatory and metabolism-regulatory features. Several lines of evidence showed that polyphenols influence some aspects of reproductive health and early development. For example, genistein reduces motility and viability of sperm, isoflavones decrease serum levels of dihydrotestosterone, while maternal consumption of polyphenol-rich foods affects fetal health [34]. However, little is known about the impact of polyphenols on placentation events beyond implantation. Most studies used a first-trimester extravillous trophoblast HTR-8/SVneo cell line with H_2_O_2_-induced oxidative damage. This cell line is shown to act as primary CTBs in response to various stimuli and represent a suitable in vitro model of human placental extravillous cells [35,36,37]. In this review, we will focus on the most investigated polyphenols and their effect on trophoblasts. Caffeic acid (CA) is a hydroxycinnamic acid, and is abundantly present in the everyday diet of pregnant women. The results obtained in animal models using CA and chlorogenic acid have shown that these two phenolic acids positively affect the rates of maturation, fertilization, and the blastocyst formation, and reduce the proportion of DNA-fragmented nuclei in oocytes after exposure to hydrogen peroxide [38]. In vitro studies on human cells showed that CA increased antioxidant capacity in endometrial cells [39] and was also efficient in inhibiting the oxidative damage in human umbilical vein endothelial cells (HUVECs) [40]. The favorable effects of CA in trophoblast cells in both normal conditions and under increased oxidative stress was confirmed in the most recent study [41]. In that study, the investigated concentrations of CA were neither cytotoxic nor genotoxic. Caffeic acid helped in the attenuation of DNA damage, protein and lipid peroxidation, and in the significant elevation of GSH concentration in human trophoblast HTR-8/SVneo cells following exposure to strong oxidant. All of these results indicate potential beneficial effects of food rich in CA for pregnancy disorders related to increased oxidative stress [41]. Besides these experiments, CA was also used for surface modification of zirconium dioxide nanoparticles (ZrO2 NPs) to attenuate NPs’ toxicity, and the results indicated that hybrid NPs did not affect viability of HTR-8/SVneo cells [42]. It has been shown that one of the most investigated dietary polyphenols, resveratrol (trans-3,5,4′-trihydroxystilbene), may also protect human HTR-8/SVneo extravillous cells against H_2_O_2_-induced OS [43,44]. Both studies showed that resveratrol ameliorates H_2_O_2_-induced cytotoxicity. It appears that resveratrol prevents OS through several steps, by restoring superoxide dismutase (SOD) and catalase (CAT) activity, by decreasing accumulation of reactive oxygen species (ROS) and malondialdehyde (MDA), and by preventing apoptosis in stressed cells. Moreover, investigation of underlying mechanisms showed that the protective effect of resveratrol on trophoblast under oxidative stress conditions is mediated by autophagic processes [43]. It was also observed that this polyphenol promotes HTR-8/SVneo cell migration/invasion and epithelial–mesenchymal transition, affecting molecules relevant for these processes [45]. Namely, resveratrol treatment increased levels of matrixmetalloproteinase (MMP)-2 and -9 and regulated expression of E-cadherin, N-cadherin, β-catenin, and vimentin. Further investigation showed that resveratrol affects the network formation ability of HUVECs, suggesting its possible role in spiral artery remodeling [46]. Like CA and resveratrol, curcumin also protects HTR-8/SVneo against H_2_O_2_-induced OS, more precisely against oxidative stress-induced apoptosis via increasing the Bcl-2/Bax ratio and decreasing the protein expression level of cleaved caspase-3 [47]. Curcumin also alleviates OS through the activation of Nrf2 signaling pathway. Recent investigation regarding its effect on functional properties of HTR-8/Svneo cells showed that curcumin positively modulates gene expression and angiogenesis, thus affecting the development of trophoblast cells [48]. Collectively, curcumin influences epigenetic modification of DNA methylation and affects cellular growth, migration, and tube formation in the human extravillous trophoblast cells.

Despite an increasing number of investigations describing the effect of different polyphenols on early-pregnancy events, studies exploring their direct actions on trophoblast still missing. Further work is required to better understand not only the antioxidant capacity of polyphenols during gestation, but also to ascertain if these molecules can be used as positive modulators of trophoblast function that may protect improper placentation.

## 3. The Role of Dietary Polyphenols in Pregnancy-Related Pathologies

### 3.1. Preeclampsia

Preeclampsia (PE) is a severe pregnancy complication affecting 2–8% of all pregnancies [49]. It is one of the leading causes of preterm birth, maternal and neonatal morbidity, and mortality worldwide [50]. According to the International Society for the Study of Hypertension in Pregnancy (ISSHP), PE is defined as de novo hypertension after 20 weeks of gestation accompanied by at least one of the following conditions: proteinuria, maternal organ dysfunction (acute kidney injury, liver dysfunction, neurological complications, hematological complications), or uteroplacental dysfunction [51]. Although the underlying causes of PE are still not elucidated completely, the etiology of PE is connected to the abnormal placentation and maternal endothelial dysfunction, leading to multiorgan disorder [49,52,53,54]. Shallow trophoblast invasion and inadequate transformation of the uterine spiral arteries, characteristic for PE, lead to the placental malperfusion, ischemia-reperfusion injury, and placental oxidative stress [52,53]. Ischemic placenta releases various soluble factors into maternal circulation, i.e., excessive amounts of proinflammatory cytokines and antiangiogenic factors, inducing damage of maternal endothelial cells and systemic endothelial dysfunction, leading to the development of hypertension, proteinuria, and other clinical symptoms of PE [52,53,54].

Strategies for PE treatment are limited to managing the symptoms pharmacologically in order to reduce maternal risk and to prolong in utero fetal development as much as possible, since ultimately, the only definitive treatment of PE is the delivery of the baby and placenta [49,51]. Although recommended antihypertensive drugs for PE management are generally considered to be safe for use in pregnancy, there is a lack of evidence-based data on long-term effects on the child of in utero exposure to these drugs [55,56]. Taking that into consideration and the limited efficiency of the existing PE treatment, there is a need for the development of new therapeutic strategies to improve both safety and efficiency of managing PE patients. Due to their antihypertensive, anti-inflammatory, antioxidative, and vasculoprotective properties, the use of dietary polyphenols could be beneficial for PE patients. Different in vitro and in vivo studies on animal PE models, as well as some clinical studies, gave promising results for using various polyphenols in PE management [57,58,59,60,61]. In mice and rat PE models, hypertension and proteinuria were alleviated by treatment with different polyphenols: resveratrol [62], curcumin [63,64], quercetin [65,66,67], punicalagin [68], and baicalin [69]. Furthermore, different PE-related adverse pregnancy outcomes in animal models, such as low fetal length and weight, low placental weight, low number of live pups, and high fetal resorption rates, were ameliorated by polyphenol treatment [63,64,65,66,67,70,71]. Beneficial effects were also noted in different organs damaged by PE-like phenotype induction on morphological and histological levels. Namely, baicalin inhibited apoptosis in kidneys and livers of PE rats inducing the expression of antiapoptotic proteins XIAP and Bcl-2 in liver cells and decreasing the expression of proapoptotic caspase-9 in liver and kidney cells [69]. In LPS-induced PE rodent models, kidneys and spleens were swollen and morphological changes in renal tissue could be noted [63,64]. Supplementation with curcumin alleviated LPS-induced injuries in those organs [63,64]. Recent preclinical and clinical studies reported enhanced efficiency of drugs usually used for PE management when combined with polyphenols [61,67,70,72,73]. Although all molecules individually showed beneficiary effects, the best results in attenuating PE-like symptoms induced in rodents were achieved when aspirin was combined with curcumin [73] or quercetin [67,70]. In clinical studies, pregnant women with severe PE who were treated with nifedipine supplemented with epigallocatechin gallate (EGCG) needed significantly shorter time to control blood pressure, had prolonged time before a new hypertensive crisis, and needed lower treatment dosages to effectively control blood pressure in comparison with a group of women suffering from PE but who were treated only with nifedipine [61]. Similar effects were achieved when PE patients were treated with nifedipine supplemented with resveratrol [72].

Imbalance in maternal circulating antiangiogenic and proangiogenic factors involved in endothelial cell damage is characteristic for PE patients [47,48,49]. In PE, maternal serum and placental levels of antiangiogenic factors, such as soluble fms-like tyrosine kinase-1 (sFlt-1) [74,75,76,77], soluble endoglin (sEng) [78,79], and endothelin-1 (ET-1) [80,81], are elevated, while proangiogenic placental growth factor (PlGF) is downregulated [75,76,82,83] in comparison with women with uneventful pregnancies. sFlt-1 is soluble vascular endothelial growth factor (VEGF) receptor 1, and binds to the VEGF and PlGF, decreasing their bioavailability and antagonizing effects mediated by these molecules [84]. In an analogous way, sEng, being its soluble coreceptor, binds to and neutralizes the proangiogenic effects of TGF-β [78,85]. ET-1 is potent vasoconstrictor peptide produced by vascular endothelial cells with an important role in the maintenance of blood pressure [86,87]. The imbalance between pro- and antiangiogenic factors in PE patients’ sera is detectable long before the onset of clinical symptoms of PE [76,79,82,83,88]. High serum sFlt-1/PlGF ratio is associated with an increased risk of PE, and it was proposed to has better predicting value than either of the biomarkers alone [89]. The beneficial effects of polyphenol treatment on the alleviation of hypertension and other PE-related symptoms in experimental animal PE models have been associated with their impact on the angiogenic factors of placental expression and maternal serum levels. Namely, quercetin supplementation decreased maternal plasma sFlt-1 and ET-1 concentrations elevated by the induction of PE-like symptoms in mice and rats in different PE models [65,66,70]. Moreover, quercetin upregulated PlGF levels in maternal plasma of PE animals [65,70]. Furthermore, the imbalance of sFlt-1 and PlGF expression in the placenta and uterus of PE animals was reversed by quercetin treatment [65,67,70]. Similar beneficiary effects was reported when mice and rats were treated with puerarin [71] and vitexin [90] in different PE models. Resveratrol showed positive effects on endothelial dysfunction induced in in vitro models [91,92,93]. This stilbenoid inhibited sFlt-1 release from human umbilical vein endothelial cells (HUVECs), placental explants, and primary cytotrophoblast and trophoblast HTR-8/SVneo cell line both under basal conditions or after stimulation with hypoxia or cytokines [91,92]. HUVECs incubated with sera of PE patients showed increased expression of endothelial dysfunction marker mRNAs, such as intercellular adhesion molecule-1 (ICAM-1), von Willebrand factor (vWF), and Caspase-3 (CAS-3) [93]. When incubated in the presence of resveratrol, mRNA expression levels were downregulated in HUVECs treated with PE sera [93]. Amelioration of PE symptoms by polyphenol treatment in PE animal models is thought to also be mediated through lowering of oxidative stress and inflammation [63,64,65,66,68,71,73,90]. Decreased oxidative stress markers, as well as increased antioxidative capacity upon treatment with different polyphenols, were reported in PE animals [65,68,90]. Polyphenol supplementation decreased proinflammatory IL-6, TNF-α, MCP-1, and other proinflammatory cytokines and upregulated anti-inflammatory IL-10 both in the maternal serum and placenta of PE animals [63,64,65,66,71,73]. According to their well-known antihypertensive, anti-inflammatory, antioxidative, and cardiovascular protective properties, it is not surprising that available studies on rodent models gave promising results for using dietary polyphenols for amelioration of PE-related symptoms. However, more studies on their mechanisms of action as well as more clinical trials are necessary to determine therapeutic effectiveness, appropriate dose ranges, and safety of using dietary polyphenols in pregnancy and as complementary therapy for PE treatment.

### 3.2. Gestational Diabetes Mellitus

Gestational diabetes mellitus (GDM) is a one of the most common pregnancy complications, defined as spontaneous hyperglycemia or the onset of any level of glucose intolerance during pregnancy [94]. Along with the worldwide increase in the prevalence of obesity and type 2 diabetes mellitus (DM), the prevalence of GDM is also rising. In Europe, the prevalence of GDM is around 11%, with the highest prevalence (31.5%) in pregnant women of Eastern European countries [95]. GDM is usually resolved following labor; however, it is associated with adverse pregnancy outcomes. There are many potential short- and long-term negative consequences of GDM for both the mother and the offspring. The risk of developing hypertension, obesity, type 2 DM, and cardiovascular disease (CVD) later in life is significantly higher in women with GDM [96]. Neonates may develop hypoglycemia, severe jaundice, and macrosomia, while long-term complications of children include hypertension, obesity, type 2 DM, and CVD [96,97,98,99]. GDM has also been reported to associate with congenital malformations [100]. Several mechanisms have been suggested for the pathogenesis of GDM, including a combination of altered adipose tissue endocrine function and placental and hormonal changes, all contributing to insulin resistance (IR). In fact, there are two main pathways leading to GDM. The first one is IR, which plays a crucial role in the pathophysiology of this condition; in normal pregnancy, it can occur due to the increased secretion of diabetogenic placental hormones [101]. It is important to stress that normal pregnancy has a diabetogenic effect on metabolism and is characterized by a state of IR, with a more than two-fold increase in insulin production to maintain euglycemia. Hence, maternal IR is physiological in pregnancy and is critically important for maintaining the maternal fuel supply to support the growing fetus, mostly during the third trimester [102]. When pancreatic β-cells cannot compensate with insulin secretion, glucose metabolism is altered, leading to GDM. Women in whom GDM develops have a significant increase in insulin response but a decrease in insulin sensitivity as the hallmarks of type 2 DM, for which they are at increased risk in later life [103]. The second pathway that leads to GDM is a chronic subclinical inflammation, and there is clear evidence that GDM is associated with changes in the maternal, fetal and placental inflammatory profile [104,105]. Furthermore, normal pregnancy is considered a state of enhanced oxidative stress that plays an important role in all stages of pregnancy, from embryo implantation, placental development, and function until parturition. It is well known that GDM is associated with a heightened level of oxidative stress, reflected through the increase in oxidative stress markers (such as lipid peroxidation index) and the decrease in antioxidative defense capacity [105,106]. Given the high prevalence of GDM and the severity of short- and long-term complications, there is an increasing demand for safe therapeutics for its prevention and treatment. Upon GDM diagnosis, diet intervention is recommended, especially for women with mild GDM, in addition to drug interventions, i.e., metformin, insulin. Based on the scientific results summarized below, polyphenols could be at least part of the answer to this important challenge. Numerous studies report the antidiabetic effects of dietary polyphenols due to their anti-inflammatory and antioxidant effects, as well as their positive effects on insulin secretion and the insulin signaling pathway [13,14,107]. The results from several studies also support the notion that some dietary polyphenols can modulate cellular and whole-body energy homeostasis under stress conditions during pregnancy, influencing AMP-activated protein kinase, the crucial cellular energy sensor [107]. A positive correlation has been found between the intake of polyphenol-rich food (PRF) and the prevention and control of cardiometabolic complications during pregnancy, including GDM [108]. The effect of polyphenol intake during pregnancy on the incidence and evolution of GDM is described recently [109]. The total intake of polyphenols, especially flavonoids, during mid-pregnancy was associated with a lower risk of GDM, according to a study on 2231 pregnant women [110], and polyphenols were proposed to be beneficial in relieving GDM symptoms. A prospective study on pregnant women with body mass index (BMI) over 30 was conducted recently. Study participants consumed two cups of whole blueberries and soluble fiber daily for 18 weeks, and it was noticed at the end of the trial that levels on antioxidant markers (reduced glutathione (GSH) and total antioxidant capacity) increased, while malondialdehyde (MDA) as a lipid peroxidation index decreased significantly [111]. Furthermore, plasminogen activator inhibitor 1 (PAI-1) decreased in maternal serum. It is known that GDM triggers the expression and release of PAI-1, which is linked with GDM severity due to excessively heightened pro-inflammatory cytokines with the development of IR [112]. Since a combination of blueberries and fiber was used in this study, it cannot be concluded with certainty what the individual contribution to this effect is, but it is likely that increased antioxidative capacity is due to blueberry antioxidants, i.e., polyphenolic compounds. Previously, the same research group showed multiple positive effects of a daily intake of two cups of blueberries with fiber for 18 weeks, beginning mid-pregnancy, in a group of pregnant women with BMI > 35 [113]. The results obtained in this randomized controlled trial suggested that investigated dietary treatment may prevent excess gestational weight gain and improve glycemic control and inflammation in obese women. Only 18% of women in the intervention group compared with 29% in the control group developed GDM. These findings surely merit further clarification through identification of active principles, most likely exerted through blueberry polyphenols. Among individual dietary polyphenols, resveratrol is the most studied one in terms of beneficial properties in GDM. A large body of evidence indicates that this antioxidant, found in red grapes and berry fruits, exerts antidiabetic action in animal models of type 2DM. Resveratrol is known to have antioxidant and anti-inflammatory properties; it improves pancreatic islet structure and function and decreases IR in animals with experimentally induced diabetes. Its effects are strongly linked to changes in expression and activity of AMP-activated protein kinase and SIRT1 in different tissues of diabetic animals [114]. Namely, AMPK is involved in the reduction of gluconeogenesis in the liver, and it has been suggested as a potential target for resveratrol. This compound reversed the glucose and insulin intolerance in the GDM mouse model, while increasing the average litter size and body weight at birth. The underlying mechanisms involved increased liver AMPK activation [115]. The regulation of the miR-23a-3p/NOV axis is another mechanism suggested according to the results obtained in the GDM mouse model [116]. Resveratrol was able to neutralize the negative effects of maternal GDM on the mouse embryos, observed through a reduction in diabetes-induced embryonic apoptosis in the cranial neural tube region and embryonic oxidative stress and improvement of antioxidant, glucose, and lipid status [117]. In the rats on high-fat and sucrose diets, maternal resveratrol supplementation, beginning at the onset of GDM in the third trimester and throughout lactation, improved glucose homeostasis and insulin secretion, without adverse effects on the offspring [118]. Du and colleagues [119] aimed to improve resveratrol stability and bioavailability through encapsulation of the resveratrol and zinc oxide complex with chitosan. These nanoparticles had no toxic effects and ameliorated diabetic signs and inflammation in the GDM rat model. In vitro, these nanoparticles exerted inhibitory effects on α-glucosidase and α-amylase activity, comparable to the antidiabetic drug acarbose [119]. In vivo rat experiments also showed that maternal diabetes may induce autism-like behavior in the offspring, in part through hyperglycemia-induced oxidative stress and SOD suppression in the amygdala. Both prenatal and postnatal treatment with resveratrol partly ameliorated this behavior [120]. In the context of inflammation associated with GDM, treatment with resveratrol significantly ameliorated the chemical and microbial induction of inflammation and insulin resistance, restored the induced defects in the insulin signaling pathway and glucose uptake, and also significantly reduced the expression and secretion of proinflammatory cytokines (IL-6, IL-1α, IL-1β) and proinflammatory chemokines IL-8 and MCP-1 in human placenta and adipose tissue [121]. Regarding the interventions with polyphenol supplements, in Malvasi et al.’s study [122], decreases in blood glucose levels and lipid profile improvement were observed in the group with trans-resveratrol supplementation. Namely, resveratrol’s addition to D-chiro-inositol and Myo-inositol treatment from the 24th to 28th week of pregnancy had beneficial effects on GDM biochemical parameters in overweight pregnant woman after 30 and 60 days. Overall, these findings support resveratrol as a dietary polyphenol that may significantly ameliorate GDM signs in pregnant women [122]. It was shown that another well-described polyphenol, curcumin, ameliorated glucose intolerance in GDM mice. It inhibited upregulated fasting blood glucose and insulin levels and restored litter size and birth weight when given orally during gestation [123]. Curcumin also attenuated oxidative stress in GDM mice through decreased lipid peroxidation marker (thiobarbituric acid reactive substances) and increased antioxidative enzyme expression (GSH, SOD, and catalase). AMPK activation, which is attenuated in GDM mice, could be restored by the treatment, thus normalizing liver glucose production through a reduction in glucose-6-phosphatase expression [123]. In a whole-mouse embryo culture model, incidence of neural tube defects induced by high glucose was lower when treated with curcumin [124]. Curcumin also suppressed oxidative stress and abolished caspase-3 and caspase-8 cleavage in this study [119], suggesting that maternal supplementation with curcumin might have embryo-protective effects in diabetic pregnant women. Both curcumin and another dietary polyphenol, phenolic acid punicalagin, possess potent anti-inflammatory properties in in vitro human models of inflammation. These compounds ameliorated TNF-induced expression of proinflammatory cytokines and chemokines, and also altered antioxidant enzymes’ (SOD and catalase) mRNA expression in placental and adipose tissue [125].

The flavonoid quercetin exhibited protective effects in several studies on GDM models [126,127,128,129]. Maternal supplementation with quercetin significantly lowered the incidence on neural tube defects and apoptosis in the mice embryos, and did so through inhibition of nitrosative stress, which is associated with increased birth defect incidence [127,129]. This effect could be exerted through inhibition of nitric oxide synthase 2, increased SOD1 expression, and decreased endoplasmic reticulum stress [127,129]. Prophylactic administration of this compound four weeks before conception resulted in an increase in blastocyst numbers and percentage of well-developed stages, and the expression of genes essential for blastocyst development and implantation as *Igf1r*, *itgav*, *itgb3*, and *COX2* [126]. Furthermore, quercetin reversed the inhibitory effect of GDM on 17β-estradiol serum level in pregnant mice. GDM led to an increase in the number of glycogen cells of the rat placenta, which was prevented by the oral administration of quercetin during gestation [128]. Quercetin also neutralized the suppressive effect of GDM on adiponectin expression and reduced the expression of its receptors [128]. This adipokine has been shown to act in a protective fashion in GDM [130]. In one word, numerous preclinical studies have shown benefits and potential of quercetin in prevention or attenuation of maternal cardiometabolic disorders, including GDM. The most recent review article highlighted the results obtained from animal studies about quercetin administration during pregnancy, emphasizing its role in modifying phenotypic plasticity [131]. Bearing in mind these results, it could be concluded that quercetin supplementation during pregnancy represents a viable strategy for changing cardiometabolic parameters throughout life. Other polyphenols have been shown to possess antidiabetic potential in GDM. One such compound is nobiletin, a flavone found in tangerine peels [132]. In in vitro tissue culture, nobiletin exerted antidiabetic activities: it improved TNF-impaired glucose uptake in skeletal muscle explants and suppressed TNF-induced proinflammatory cytokines and chemokines in the placenta [133]. These observations were verified in vivo in a mouse GDM model. GDM mice treated with nobiletin, either in a prophylactic or treatment manner, significantly suppressed proinflammatory cytokines and chemokine expression and secretion in placenta and adipose tissue. Nobiletin holds a great promise for the prevention/treatment of GDM, given that supplementation of either citrus flavonoids or Diabetinol^®^ (with nobiletin as the primary active ingredient) has already been found to significantly improve fasting glucose and lipid profiles in nonpregnant diabetic subjects [134,135]. Epigallocatechin gallate, a flavanol abundant in green tea, given orally to pregnant diabetic mice, significantly decreased dam neural tube defect formation induced by high glucose and inhibited maternal diabetes-induced global DNA hypermetylation, as well as DNA methylation in the CpG islands of genes essential for neural tube closure [136]. Oleuropein, a dietary polyphenol present in olive fruit and oil, was investigated in a recent study conducted by Zhang et al. [137]. It attenuated the elevated body weight of GDM mice; gestational outcome was markedly improved. Due to oleuropein administration, markers of oxidative stress and inflammation as well as blood glucose, insulin, and hepatic glycogen levels were alleviated, and the AMPK signaling pathway was activated. Pomegranate ellagic polyphenols (PEP) exerted beneficial effects in a rat model of GDM, which was reflected in restored body weight and fetal body weight, and normalization of fasting glucose, insulin levels, and insulin resistance index [138]. Apart from beneficial effects for the lipid status, PEP reversed the negative effects of GDM on 11β-hydroxy steroid dehydrogenase type 2, an enzyme that protects the fetus from excessive cortisol in utero, as well as proteins implicated in insulin resistance. The inflammatory mediators TNF-α, IL-6, and CRP were also lowered by the treatment. The findings of that study also indicate that PEP could regulate the PPARα-TRB3-AKT2-p-FOXO1-GLUT2 signaling pathways, which are related to insulin sensitivity [138].

In conclusion, dietary polyphenols possess numerous antidiabetic effects, including increasing glucose tolerance and improving lipid status, while decreasing inflammation, oxidative and endoplasmic reticulum stress, and lowering GDM-induced birth defect incidence. Additional efforts should be put into the careful design of polyphenol-rich diets and single-polyphenol supplementation for GDM prevention or treatment. Potential adverse effects and organ toxicity should be carefully assessed.

## 4. Potential Harmful Effects of Dietary Polyphenols in Pregnancy

Despite the abundant evidence of polyphenol’s beneficial antioxidative and anti-inflammatory effects on reproductive health [34], little is known about the wide range of their biological actions in pregnancy, and the short- and/or long-term effects they might exert on offspring health. Data regarding the influence of polyphenolic compounds on oocyte maturation, fertilization, and development of the blastocyst are still conflicting. When analyzing the effects of polyphenols on pregnancy processes, it should be taken into account that interventions in early physiological inflammatory and redox signaling may pose a significant risk for both maternal and offspring health. Namely, moderate levels of ROS support early pregnancy through the activation of several redox-sensitive pathways to promote decidualization, embryogenesis, attachment of the embryo to the uterine wall, trophoblast invasion, neaoangiogenesis, and vascular remodeling in the placenta [31,139,140]. Consistently with these notions, reducing ROS production during placentation impaired trophoblast invasion and placental development in mice, leading to the development of PE symptoms [141]. This could be related to impaired differentiation of proliferative CTB to invasive EVT cells, by altered expression of α1/β1 integrins on CTB, inhibited activation of MMPs in EVT cells, and other mechanisms proposedly mediated by ROS [29,30,142,143]. The establishment of pregnancy is also driven by locally synthesized inflammatory cytokines and prostanoids from the decidual endometrium [144,145]. The major pharmacological action of polyphenols on gestational processes might come from interference in the inflammatory cascade, by inhibition of cyclooxygenase (COX)-2 activity and the transformation of arachidonic acid into PGs [146]. These inhibitory effects of polyphenolic compounds on PG synthesis in maternal reproductive tissues and the fetus are thought to be analogous to the effects of nonsteroidal anti-inflammatory drugs (NSAIDs) [147]. Apart from arachidonic acid pathways, plant polyphenols are documented to negatively regulate the nuclear factor kappaB (NF-κB) signaling pathway [148,149]. In the same vein, selected phytochemicals have shown to inhibit inflammasome activity in in vitro and in vivo tests [150]. Targeting NF-kB and inflammasome activity, polyphenolic use may result in transcriptional repression of a large number of inflammatory genes, including those encoding TNF-α, IL-1β, IL-6, IL-12p40, 17A, and COX2; the downregulation of chemokines; and decreased generation of ROS [151,152,153,154]. Considering the aforementioned, it is reasonable to hypothesize that the anti-inflammatory, antioxidative, antiproliferative, and vasoactive properties of polyphenols could interfere with blastocyst development, implantation, and postimplantation processes. This hypothesis has been supported by numerous results from in vitro and animal models. For instance, genistein treatment of murine oocytes significantly reduced the rate of oocyte maturation, in vitro fertilization, and embryonic development [155]. Similarly, treatment of murine oocytes with high-dose curcumin during in vitro maturation (IVM) impaired blastocyst development from the morula, promoted early-stage death of mouse blastocysts, increased resorption of postimplantation embryos, and decreased fetal weight [156]. Moreover, in mice, maternal intake of dietary curcumin in higher concentration significantly decreased in vivo oocyte maturation and fertilization, and inhibited embryonic development from the zygote to blastocyst stage [156]. It also reduced the potential of implantation and postimplantation development [156]. Embryotoxic effects of curcumin at the early postimplantation stage of gestation were confirmed in another study, where exposure to 24 μM of curcumin at the blastocyst stage was lethal to all embryos [157]. Curcumin cytotoxic effects were suggested to stem from improper activation of apoptotic processes through increased Bax and reduced Bcl-2 expression, ROS generation, and caspase-3 activation in early-stage embryos, also suggesting its role as a teratogen [158]. Administration of high-dose quercetin reduced the expression and distribution of uterine receptivity molecules (mucin-1, E-cadherin and integrin αVβ3) during the peri-implantation period in rats [159]. It also increased estrogen levels, whereas it decreased progesterone in early-pregnant rats, possibly through interference with the enzymes involved in sex steroid biosynthesis [159]. Additionally, quercetin at high doses exhibited severe teratogenic effects, causing neural tube defects, somite dysmorphology, and telencephalic hypoplasia, among other injuries, in IVM mouse embryos [160]. Similarly, naringenin [160] and Ginkgolide B of ginkgo biloba extract [161] induced growth retardation, developmental defects, and reduced viability in cultured mouse embryos. Still, polyphenolic-induced embryotoxicity or failure of early-pregnancy endpoints was not shown in other rodent studies [162,163,164]. Furthermore, curcumin, quercetin, and naringenin applied at lower doses than those exhibiting embryotoxic effects were suggested to have protective effects against other teratogenic agents [160,165]. These inconsistencies may reflect differences between the studies in terms of animal species- and strain-specific susceptibility, and the experimental design (timing and dosing regimes, etc.) used in the studies. Due to medical and ethical constraints in research on very early-pregnancy endpoints in humans, studying women undergoing in vitro fertilization (IVF) remains the only means of observing the impact of dietary polyphenols on early developmental measures. One such study revealed that resveratrol supplementation in women undergoing IVF was strongly associated with lower implantation and pregnancy rates and higher miscarriage rates [166]. One of the proposed mechanisms behind this outcome is thought to be resveratrol’s anti-inflammatory action and direct inhibition of embryo attachment [166]. Resveratrol use was associated with antideciduogenic effects by modification of genes regulating decidualization and suppressing decidual senescence [167], which could increase the risk of implantation failure and miscarriage. Furthermore, a dramatic increase in fetal pancreatic mass and exocrine cell proliferation following maternal resveratrol supplementation in nonhuman primates was shown [168]. Considering that resveratrol-rich grape juice and derivatives are consumed daily worldwide by pregnant woman, further studies about possible harmful effects of resveratrol use during human pregnancy should be conducted. Besides early reproductive toxicity, PRF use during pregnancy could also affect developmental trajectories, causing miscarriage or preterm delivery [169,170]. Feverfew tea, traditionally used for the treatment of fevers and headache, is also a potent abortifacient (which is another traditional use), and is contraindicated in pregnancy [171]. Parsley, pennyroyal, calendula, saffron, knotweed, nutmeg, and many other polyphenol-containing plants have also been associated with a risk of miscarriage [172,173,174]. Moreover, plant products containing isoflavonoids or thiocyanate, such as green tea, lemon balm leaves, and soy beans, are suggested to inhibit thyroperoxidase and/or tissue deiodinases, leading to early maternal hypothyroxinemia that may induce severe neurological damage during the sensitive period of neuronal cell migration [175]. Owing to their anti-inflammatory and antioxidant properties, polyphenol consumption during pregnancy has been commonly associated with a serious vascular malformation, premature closure of ductus arteriosus (PCDA) [147]. Several experimental and clinical studies have reported PCDA after a history of maternal abundant polyphenol consumption. For instance, the use of green tea [176], matè leaf herbal tea infusion [177], or chamomile tea [178], daily intake of prune berry and violet vegetable juice (a blend of 18 vegetables and nine fruits that contains anthocyanins) [179] or MonaVie (a juice blend containing the cyclooxygenase and nitric oxide synthase inhibitors, proanthocyanidins and anthocyanins) [180], or excessive quantities of fresh oranges [181] and dark chocolate [182] in late pregnancy have all been associated with PCDA. The underlying pathogenetic mechanisms are thought to be similar to those involved in PG inhibition by NSAIDs [183]. Additionally, analogue to the effects of NSAIDs, PRF-induced PCDA was ameliorated or completely reversed when these substances were discontinued [181,184]. Functional closure of the ductus in healthy term newborns occurs within the first postnatal days, while complete fibrous obliteration develops after several weeks [185]. This is a result of a well-balanced interplay between locally produced and circulating mediators, of which PGs have a major role, and the particular structure of the vessel wall [186]. As both COX-1 and COX-2 are expressed in endothelial and smooth muscle cells of the ductus arteriosus [187,188], prenatal inhibition of their activity may cause constriction, or premature closure of the ductus [188]. Polyphenols are also shown to induce PCDA by inhibiting NO-mediated vasodilatation in pregnant sheep after PRF intake for 14 days [189]. The ductus arteriosus vulnerability to constrictive factors, thus the risk of PCDA, progressively increases with gestational age (e.g., the sensitivity to indomethacin-induced vasoconstriction is approximately 5–10% at 27 weeks, 15–20% at 32 weeks, and almost 100% at 34 weeks [190]. To date, there is no consensus concerning the amount of polyphenol consumption that could cause PCDA. The evidence already available warrants caution regarding PRF consumption in the third trimester of pregnancy, due to increased risk of PCDA and its pathophysiological consequences [147]. Current evidence of the metabolism and pharmacokinetics of polyphenols ingested in pregnancy indicates that the transplacental transport of these substances may potentially interfere with fetal developmental processes [191,192]. The concept of “fetal programming” defines fetal adaptive responses to the conditions encountered in utero, which results in various (mal)adaptations that may be disadvantageous in adult life. Epigallocatechin-3-gallate, caffeic acid, catechin, curcumin, epicatechin, lycopene, genistein, quercetin, resveratrol, rosmarinic acid, and other dietary polyphenolic compounds, which are an integral part of human diet, are thought to be involved in fetal programming by inducing changes to the fetal epigenome [193,194,195]. These changes to the structure of DNA without changing the underlying DNA sequence may have long-lasting effects, as they may be transmitted to daughter cells [196,197,198], and down to the offspring via transgenerational epigenetic inheritance [199].

In conclusion, despite the growing body of studies highlighting potential benefits, unambiguous links between polyphenols and their safe use in pregnancy are insufficient and there is still a lack of evidence-based data. As in vitro animal and human studies suggest, dietary polyphenols, especially in excessive dosages, may have adverse effects on fetal health and pregnancy outcome. They have been shown to influence early gestational process by disrupting endometrial receptivity, embryo development/survival, and implantation and placentation processes [159]. They might also interfere in subsequent fetal developmental trajectories through epigenetic changes, with possible lifelong and/or transgenerationally altered expression of certain genes [195]. By skewing the intrauterine inflammatory and redox environment, overconsumption of PRF in the third trimester may induce fetal/newborn defects such as PCDA and the related consequences [147]. All the aforementioned warrants particular attention, considering (i) the increased supplement consumption by pregnant woman [200], and (ii) that polyphenol intake through supplements is thought to be approx. 100 times greater than through a Western diet [201]. Additionally, some unexpected effects of PRF may be influenced by various individual factors, such as women’s age, genetics, nutrition status, comorbidities, concomitant use of other bioactive substances, etc. All of these aspects should be taken into account when designing future studies aimed at improving our understanding of the mechanisms of functional foods and their safety in pregnancy.

## 5. Conclusions and Future Research Directions

It is well documented that a polyphenol-rich diet has valuable health benefits and can be considered as a powerful tool for the prevention of numerous chronic diseases; recently, their effects have been also underlined against coronavirus disease 2019 (COVID-19) [202,203]. Dietary polyphenol consumption during pregnancy requires particular attention considering the potential influence of polyphenols on the mechanisms of maternal adaptation and fetal development. Both the potential health benefits and the possible adverse effects should be considered when it comes to the consumption of dietary polyphenols during pregnancy.

Currently, native polyphenols found in food are the most often investigated polyphenol compounds in vitro. However, polyphenol metabolites might be the biologically active compounds rather than simple polyphenols. Hence, further in vitro studies are required to show the biological activity of polyphenol metabolites. A crucial aspect that should be implemented in the design of future studies on the topic is the clinical relevance of in vitro findings. Namely, current studies are often conducted using polyphenol concentrations that may exceed normally found physiologic concentrations [34]. Little is known about the role of dietary polyphenols in advanced maternal age. This topic could be also significant for future research, as maternal aging creates a suboptimal environment for placental and fetal development that contributes to the vulnerability to adverse outcomes [204]. Another underinvestigated area in the field is the influence of polyphenols present in prenatal nutrition on the long-term maternal health as well as the metabolic risk for the offspring, or in one word, in transgenerational health promotion. Clarification of all these points is essential for issuing future dietary polyphenol guidelines during pregnancy.

## Data Availability

Not applicable.
